# Connectome of a human foveal retina

**DOI:** 10.21203/rs.3.rs-7312705/v1

**Published:** 2025-08-20

**Authors:** Yeon Jin Kim, Orin Packer, Thomas Macrina, Andreas Pollreisz, Christine A. Curcio, Kisuk Lee, Nico Kemnitz, Dodam Ih, Tri Nguyen, Ran Lu, Sergiy Popovych, Akhilesh Halageri, J. Alexander Bae, Joseph J. Strout, Stephan Gerhard, Robert G. Smith, Paul R. Martin, Ulrike Grünert, Dennis M. Dacey

**Affiliations:** 1 Department of Neurobiology & Biophysics, University of Washington, Seattle, WA, 98195; 2 Zetta AI LLC, Sherrill, NY, USA; 3 Department of Ophthalmology, Medical University of Vienna, Vienna, Austria; 4 Department of Ophthalmology and Vision Sciences, University of Alabama at Birmingham, Alabama 35294; 5 Aware LLC, Rappenstrasse 19, 8307 Effretikon, Switzerland; 6 Department of Neuroscience, University of Pennsylvania, Philadelphia, PA 19104; 7 The University of Sydney, Save Sight Institute and Discipline of Ophthalmology, Faculty of Medicine and Health, Sydney, NSW, 2000, Australia; 8 Washington National Primate Research Center, Seattle, WA 98195

## Abstract

What makes human brains distinctive? The answer is hidden at least partially in the myriad synaptic connections made between neurons – the connectome. The foveal retina is a primate specialization which presents a feasible site for deriving a complete connectome of a human CNS structure. In the fovea, cells and circuits are miniaturized and compressed to densely sample the visual image at highest resolution and initiate form, color and motion perception. Here we provide a draft connectome of all neurons in a human fovea. We found synaptic connections, distinct to humans, linking short-wavelength sensitive cones to color vision pathways. Moreover, by reconstructing excitatory synaptic pathways arising from cone photoreceptors we found that over 95% of foveal ganglion cells contribute to only three major pathways to the brain. Our study reveals unique features of a human neural system and opens a door to a complete foveal connectome.

The vertebrate retina, an accessible central nervous system (CNS) outpost where the visual process starts, has long been appreciated for its great complexity^1^ and for linking neural structure to visual function^2–4^. The retina was also an early focus of synaptic level connectomic reconstruction^5–10^, and more recently, retinal cell populations have also been linked to their molecular profiles^11–14^ and the genetics of human disease^13, 15^. Yet even for this intensively studied neural system, we lack a full wiring diagram that could explain how one of our most fundamental senses – vision – is initiated.

The recent achievement of a complete connectome of the brain of the fruit fly, Drosophila melanogaster^16, 17^, has made the connectome of a larger mammalian brain a now-conceivable goal^18–21^ and connectomic methods have recently been applied to mm-scale volumes of mouse and human neocortex^19, 20, 22, 23^. In the present study we take advantage of the distinctive neural architecture of the foveal retina, to provide a first draft connectome of this critical locus in the human CNS.

The fovea is a specialized central region in the retina of humans and other day-active primates where neural circuits are spatially compressed to facilitate high resolution form, color and motion perception. At barely 1 mm wide the fovea encodes only a few degrees of visual space, but its representation is magnified 100-fold across the surface of primary visual cortex^24–28^. Accordingly, the fovea is essential for normal vision, and failure of foveal function is a hallmark of devastating blinding retinal diseases.

From a signal-processing viewpoint, the fovea is a self-contained input-to-output sensory structure, where light transduction by photoreceptors is processed by diverse neural cell types across two narrow cell and synaptic layers. Here we provide a catalog of human foveal cells as well as assignment of synaptic connections to distinct functional circuits. We use this dataset to directly address the long-standing question of the number and identity of visual pathways that link the fovea to the brain. We find only three visual pathways account for over 95% of the eye’s projection to the human brain: a striking reduction compared to both non-primate mammals^29–31^ and non-human primates^32, 33^. In addition, we observed unexpected differences in synaptic connections compared to non-human primates and provide evidence that neural coding for trichromatic color vision begins with circuitry that is uniquely human.

## Results

### Acquisition of the foveal volume.

**Fig. 1.**
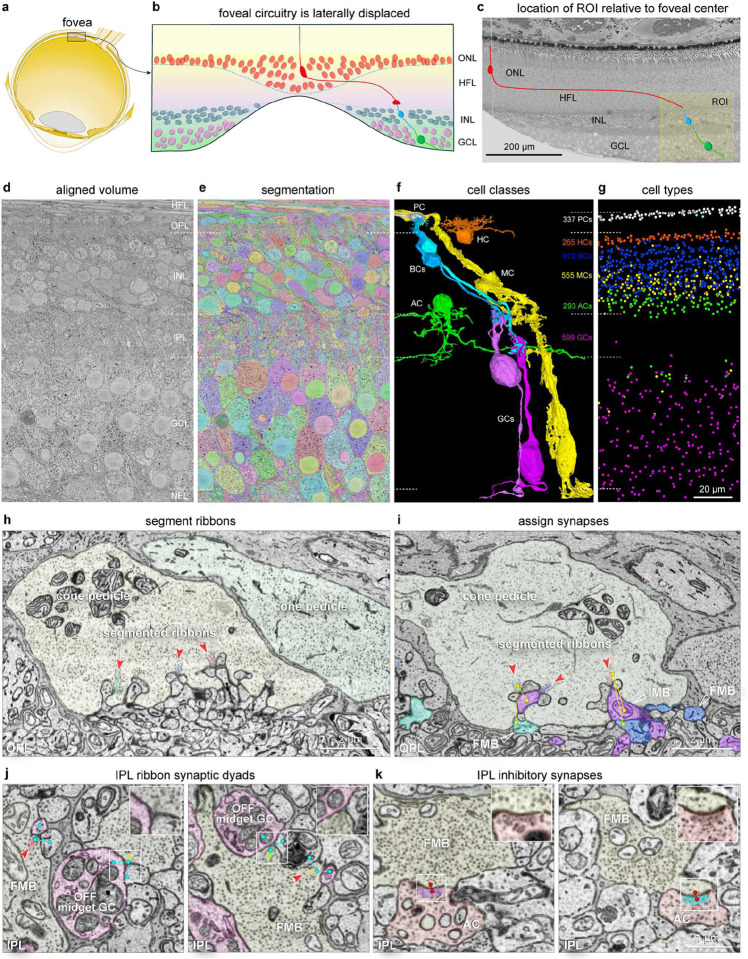
The HFseg1 volume. **a.** The fovea forms a pit in the retina ~3 mm temporal to the optic disc (within the inset rectangle). **b**. Schematic foveal pit showing cone (red) to bipolar cell (blue) to ganglion cell (green) circuit. ONL, outer nuclear layer, HFL, Henle fiber layer; INL, inner nuclear layer; GCL, ganglion cell layer. **c**. Trimmed retinal block face and ROI (yellow box) in temporal retina 400–500 μm from the foveal center. **d**. Single section from the imaged volume (cropped laterally) to illustrate the retinal layers, HFL, OPL, outer plexiform layer; INL; IPL, inner plexiform layer, GCL and NFL, nerve fiber layer. **e**. Volume segmented into membrane bound objects. **f**. Examples of segmented cells photoreceptor axon terminal (PC, white), horizontal cell (HC, orange), ON and OFF bipolar cells (BC, light and dark blue), Müller glia cell (MC, yellow), amacrine cells (AC, green), ON and OFF ganglion cells (GC, dark and light violet); astrocytes and microglia not shown here. **g**. Numbers and cell soma locations of the cell types shown in **f**. **h.** Three segmented ribbons (varied colors) at synaptic triads (red arrowheads) in a single cone pedicle. **i.** Synaptic assignments associated with each ribbon (yellow dots and lines). An IMB (inner-ON midget bipolar; violet, white open arrowhead) cell makes an invaginating contact and two FMB cells (outer-OFF midget bipolar; light and dark blue, white open arrowheads) make a basal contact with the cone pedicle. **j.** Examples of ribbon and synapse prediction in the inner plexiform layer (IPL) for an FMB cell axon terminal at two locations (left and right panels). Segmented ribbons (varied colors, red arrowheads) and synaptic predictions (blue dots, lines). **k.** Synaptic vesicle cloud segmentation (varied colors) and synaptic prediction (red dots and lines) in the IPL between an amacrine cell and an FMB axon terminal. Insets in **j** and **k** show zoomed view of synapses indicated by white boxes.

Our goal was to obtain complete retinal circuitry near the peak of cone density (peak of visual acuity)^34, 35^. This is a difficult task because (among other reasons) foveal cones feed inner retinal circuits via very long axons (Henle fibers, Fig. 1a–1c)^36^. Our data sample therefore was centered at 500 μm retinal eccentricity, where cone axon length averages 300 μm^37^; this location is linked to cones located ~200–300 μm from the center of the foveal pit (~0.7–1 deg). We cut 3028 vertical sections at 50 nm through a depth of ~150 μm and imaged an area that was 180 μm tall × 180 μm wide at 5 nm resolution (voxel size, 5×5×50 nm^3^), extending from the Henle fiber layer (outer retina) to the nerve fiber layer (inner retina) (Fig. 1d). Recently developed computational methods (convolutional neural networks)^38, 39^ were used to align the images, detect membrane boundaries, segment neurons, glia, blood vessels, simple (conventional) and complex (ribbon) synapses, and predict synaptic relationships in the outer and inner synaptic layers (Fig. 1d–1k; see Methods for procedures). For ease of reference, we named this volume HFseg1 (Human Fovea, segmented 1). All annotated cell types and the draft catalog of synaptic connections are available for exploration using search tools in an openly accessible form (https://neuromaps.app/datasets, see Supplementary Data Table 3) which utilizes the Neuroglancer browser and CAVE infrastructure. In addition, we have created a second site (accessed via NeuroMaps) that facilitates directed proofreading of both cells and synapses by a user community.

### Cell populations

To derive and understand any connectome, the identity and layout of all cell types must first be clearly understood. Here, we took advantage of a well-established morphology of the majority of foveal cell types^40, 41^ that have also been linked to transcriptomically defined cell clusters in both human and non-human primate retina^12, 13, 42^. Moreover, for many cell populations the presence of orderly spatial arrays and their distinctive morphology could be used to define not only a cell type but its relative density. We were therefore able to use the original segmentation followed by proofreading of cell morphology (~37,000 edits to date) to annotate and classify all cells by class and specific type and to assess the quality of synapse prediction (Fig. 1f–1g; see Supplementary Data Table 1a, 3008 total cells with annotated cell bodies and ~368,000 total synapses).

**Fig. 2.**
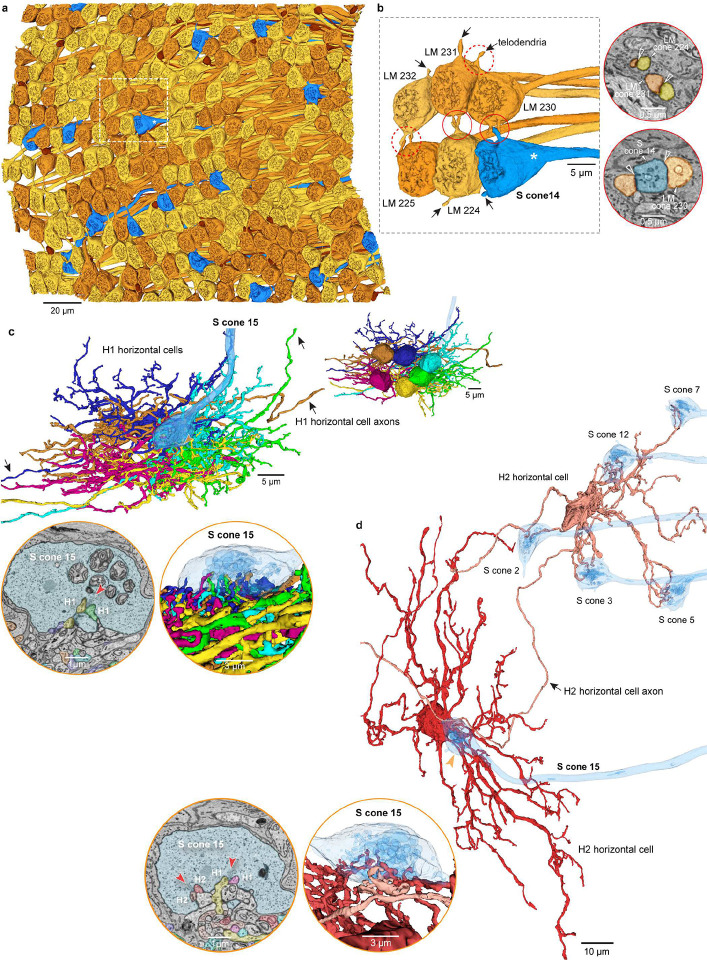
Photoreceptor and horizontal cell populations. **a**. Array of photoreceptor axon terminals (cone pedicles, rod spherules) viewing the synaptic face of the terminals (297 LM cones, gold; 16 S cones, blue, 24 rods, brown) (see Supplementary Data Table 1a, 1c, 1d). **b**. Six cone pedicles within the white dotted box in **a**. Left, the pedicles contact each other via telodendria (e.g., arrows) within the circled areas. A pedicle identified as an S cone (white asterisk) contacts neighboring LM cone 230. Right, EM micrographs showing telodendritic contacts (open white arrowheads) between LM cones (top) and S cone with LM cone (bottom). **c**. Six overlapping H1 cells are shown (varied colors); each H1 cell forms lateral elements in S cone 15 (blue). Upper inset shows the cell bodies of the six H1 cells. Circle inset at lower left shows EM view of H1 cell lateral elements at a synaptic invagination of S cone 15 (red arrowhead). Circle inset at lower right shows a 3D view of S cone 15 (partial transparency) contacting multiple H1 cells. **d**. Two H2 cells (tan and red) contacting multiple S cones, including S cone 15. Lower insets show H2 cell lateral elements at S cone 15 in single layer EM view (left) and 3D (right) view (conventions as in **c**).

**Fig. 3.**
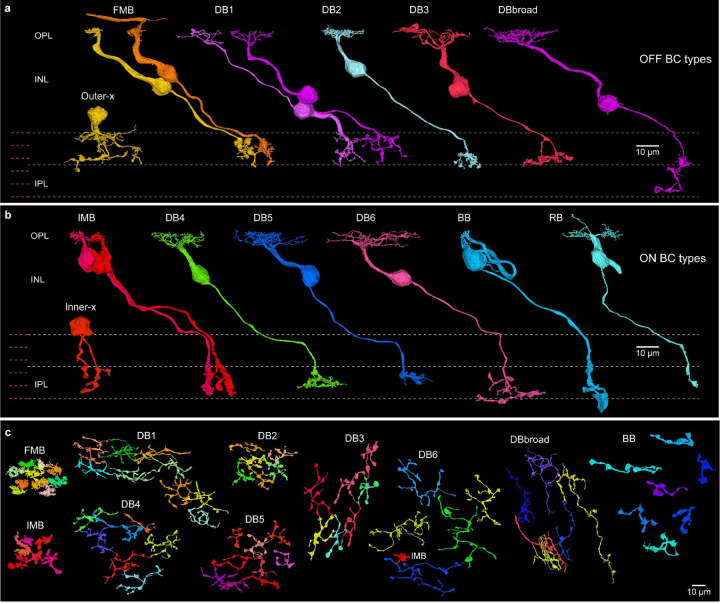
Bipolar cell types. **a**. Outer stratifying types (FMB, flat midget bipolar; DB, diffuse bipolar). Outer-x bipolar cells lacked a dendrite but made ribbon synapses in the IPL (Extended Data Fig. 6a). DBbroad cells extend processes across the IPL depth (Extended Data Fig. 7). **b.** Inner stratifying bipolar types; IMB, invaginating midget bipolar; DB, diffuse bipolar types; BB, blue-cone bipolar type, Inner-x bipolar (Extended Data Fig. 6c); RB, rod bipolar. **c**. Horizontal view of spatial mosaics of axon terminals for small groups of neighboring cells of each bipolar type, as indicated.

In brief, HFseg1 contained 313 cone (pedicles) and 24 rod (spherules) synaptic terminals (rod: cone = 0.07, Supplementary Data Table 1c, 1d). Sixteen cone pedicles were identified as short wavelength sensitive (S) cones (Supplementary Data Table 1c; Fig. 2a)^43^. The remaining 297 cones comprised long (L) and medium (M) wavelength sensitive cones, here referred to collectively as LM cones. Postsynaptic to cones were 268 horizontal cells and 912 bipolar cells (Supplementary Data Table 1e, 1f). It was possible to distinguish the H1 and H2 horizontal cell types by distinctive morphology and connectivity (Fig. 2) and most of the classically recognized bipolar cell types (midget and diffuse bipolar types). Some exceptions will be considered further below (Fig. 3).

**Fig. 4.**
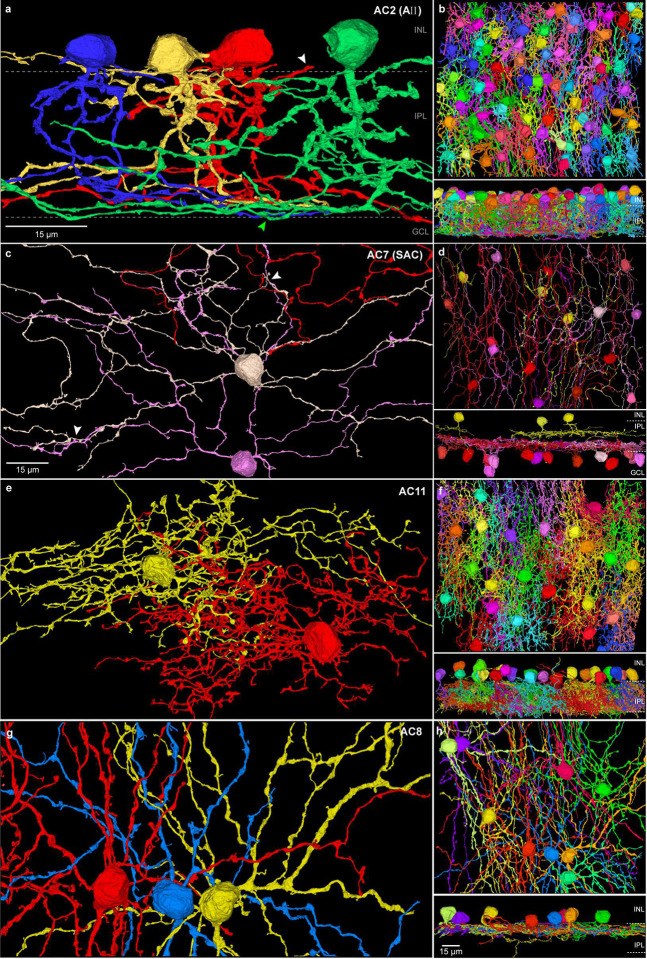
Amacrine cells. Amacrine cells in the INL (253 cells) and GCL (40 cells) were divided into 19 types (AC1–19; Supplementary Data Table 1g). **a.** Small field, AII amacrine cells (AC2), in vertical view show outer, lobular dendrites (white arrowhead) and extensive, inner dendrites (green arrowhead). **b.** Horizontal view of the entire AII population in HFseg1; lower panel, vertical view. **c.** Starburst amacrine cells (AC7) were located primarily in the GCL (GCL, 14 cells; INL, 3 cells). White arrowheads indicate typical fasciculation of starburst dendrites. **d.** Mosaic of starburst cells, GCL cells (red-violet) and INL cells (yellow); lower panel, vertical view. **e**. Small field amacrine type (AC11) in horizontal view with densely branched dendritic arbor. **f**. Horizontal view of the AC11 cell mosaic; lower panel, vertical view shows dendrites extend broadly across the IPL. **g.** Large field amacrine cells (AC8) with thick lobular dendrites, narrowly stratified in the outer IPL. **h.** Horizontal view of the complete mosaic; lower panel shows vertical view of outer IPL stratification. Scale bar in **c** applies to **e** and **g**. Scale bar in lower panel of **h** applies to **b, d, f**.

**Fig. 5.**
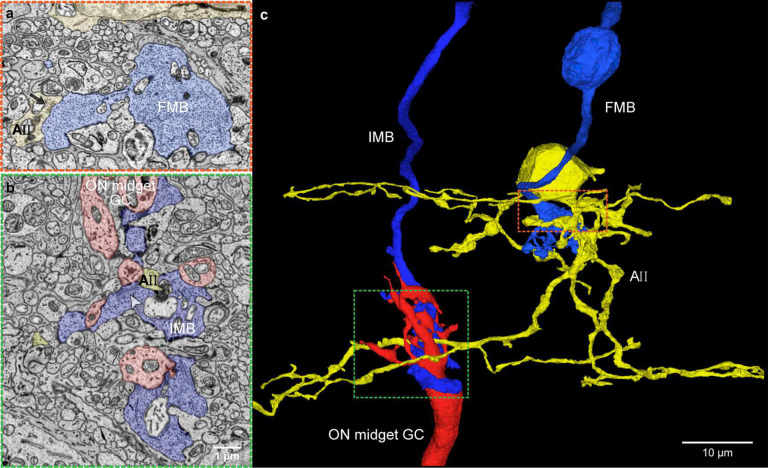
AII amacrine cells are connected to the ON and OFF midget circuit. **a.** The AII cells provide abundant inhibitory synaptic output to FMB (OFF-midget bipolar) cells (black arrow and orange boxed area in **c**). **b.** Rod bipolar cells are largely absent in HFseg1 volume. This AII cell (yellow) receives alternate excitatory synaptic input from IMB (ON-midget bipolar) cells (white arrowhead and green boxed in **c**). **c.** Vertical 3D view; AII cell shown in yellow. FMB and IMB cells shown in blue. ON midget ganglion cell in red; OFF midget ganglion cell not shown in this image.

We annotated 293 amacrine cells, including 40 cells displaced to the ganglion cell layer (Supplementary Data Table 1g, Figs. 4, 5; Extended Data Figs. 6, 8), first dividing cells by dendritic field diameter into small (diameter < 100 μm; n = 189), and large field (diameter >100 μm) groups (n = 104). We divided these groups in turn into 19 types (6 small field and 13 large field; Supplementary Data Table 1g) based on morphology, stratification, mosaic interactions and nuclear morphology (Extended Data Figs. 3, 6, 8). Figure 4 provides a partial view of this dataset by highlighting four cell types (see also Supplementary Data Table 3); two additional amacrine populations are illustrated in Extended Data Figure 8 and others can be viewed at the NeuroMaps site.

We annotated 599 ganglion cell bodies (Fig. 1g; Supplementary Data Table 1h). As expected, the classically recognized midget GCs constitute the great majority (536 cells, ~90%, Supplementary Data Table 1h)^13, 44^; these were readily divided into outer-OFF stratifying (280 cells) and inner-ON stratifying (256 cells) types (Fig. 7a, 7e). A second well defined cell group, the parasol cells (26 cells, 13 inner-ON cells and 13 outer-OFF cells, ~4% of total GCs) was also easily distinguished (Fig. 7b, 7f; Supplementary Data Table 1h). Small bistratified GCs^45^, found to show blue-yellow color opponency in macaque retina^46, 47^ were distinguished by their broad dendritic ramification (12 cells, ~2% of total GCs, Supplementary Data Table 1h; Fig. 7c, 7g) and extensive synaptic input from blue cone bipolar cells (BB cells)^48^. Together, the midget, parasol and small bistratified cells accounted for 95.8% of the ganglion cells in HFseg1 (Supplementary Data Table 1h). The remaining 25 ganglion cells showed relatively large dendritic fields and were divided provisionally into five types (Supplementary Data Table 1h, Supplementary Notes) pending a more detailed analysis of their synaptic connections.

Lastly, glial cells were readily distinguished from all neurons by their distinctive ultrastructure (Extended Data Fig. 3a, 3b, 3c, 3f). The Müller cells (555 cells, Fig. 1g; Supplementary Data Table 1b) were distinguished as radial glia with cell bodies in the inner nuclear layer (Extended Data Fig. 1a). A second, morphologically distinct population of glial cells in the GCL was closely associated with the superficial blood vessels there (Extended Data Figs. 1b and 3b) and provisionally identified as astrocytes^49^ (32 cells, Supplementary Data Table 1b; Extended Data Fig. 1b). We also identified 15 microglial cells (Supplementary Data Table 1b) with cell bodies in the IPL or GCL (Extended Data Fig. 1a) distinct from both Müller cells and astrocytes (Extended Data Figs. 1a, 1b and 3c, 3b; Fig. 1f). The density of microglial cells was low, consistent with previous measurements in the macaque monkey foveal retina^50, 51^. In the current volume we have not yet attempted to characterize blood vessel and associated pericytes, though these cellular elements are also segmented and are available to annotate and further characterize. More detailed information on cell type identification is provided in a supplement (Supplementary Notes on cell type identification).

### Synapse detection

To assess the accuracy of ribbon segmentation and synapse prediction in HFseg1 (Fig. 1h–1k) we compared deep-learning predicted synaptic assignments to manual (ground truth) assignments. We focused on the cone-to-midget bipolar cell synapse in the OPL and the midget bipolar cell synapses to amacrine and ganglion cells in the IPL. For ground truth, we manually annotated all synaptic connections for the midget pathway connections to three LM cones (cones 21, 22, and 58; Extended Data Fig. 9; Supplementary Data Table 2a-2c).

**Fig. 6.**
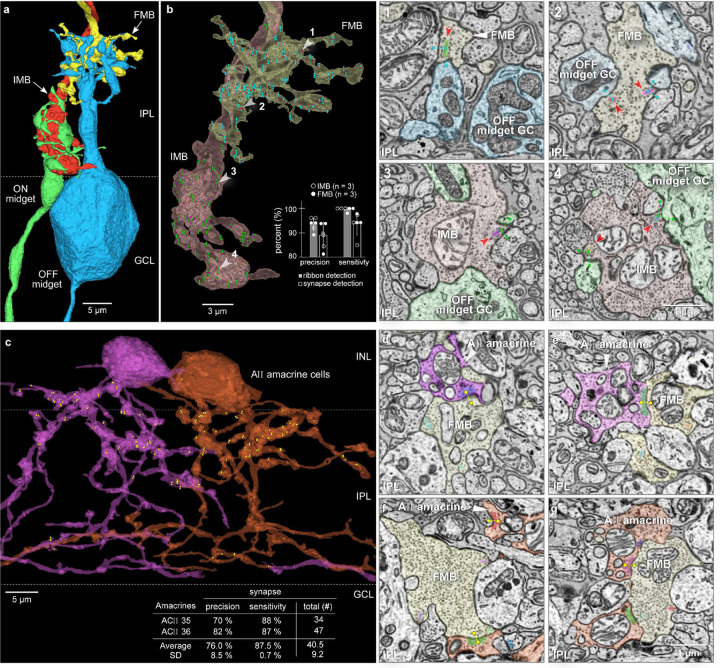
Synapse detection. **a**. OFF midget bipolar (yellow) and ON midget bipolar (red) presynaptic to an outer-OFF (blue) and inner-ON (green) midget ganglion cell. **b.** Outgoing synapses detected for the midget bipolar cell axon terminals shown in **a** (ganglion cells removed, semi-transparent view). Synaptic ribbons (red/violet dots) and postsynaptic profiles were first identified manually (see Extended Data Fig. 9; cone 22; BC 308 (IMB) and BC 303 (FMB)). Predicted outgoing synapses (green dots and lines, IMB; blue dots and lines, FMB). White arrowheads numbered 1–4 indicate the panels shown on the right in single layer EM view. Predicted and segmented synaptic ribbons (in varied colors, red arrowheads); predicted postsynaptic segments are marked by the dots and lines linking the synaptic ribbon to its postsynaptic structure (unlabeled postsynaptic profiles belong to amacrine cells). Inset histogram shows precision and sensitivity of ribbon detection and synaptic assignment. **c.** Inhibitory output synapses made by two neighboring AII amacrine cells (purple and orange in partial transparency). Yellow dots show synaptic output predictions; most synaptic outputs arise in the outer half of the IPL and are presynaptic to OFF bipolar cells. Inset shows precision and sensitivity for the synapses made by each AII cell. **d-e**. Single layer images showing segmented vesicle clouds (varied colors) in the purple AII cell (shown in **c**) and synaptic connections predicted with an FMB cell (yellow dots and lines; ribbons in the FMB are also segmented). **f-g**. Same conventions as in **d-e** but showing three segmented vesicle clouds and synaptic predictions for the orange AII cell (shown in **c**), with another FMB cell.

Figure 6 shows the predicted outgoing synapses for a pair of midget bipolar cell axon terminals. Measures of precision (true synapses / [true synapses + false positives]) and sensitivity (true synapses / [true synapses + false negatives]) were taken for ribbons and their synapses. The segmentation of ribbons showed precision and sensitivity near 100% (n = 370 ribbons; n = 6 midget bipolar cells; precision: 93.5% ± 2.7%; sensitivity: 99.7% ± 0.8%, Fig. 6b inset). Post-synaptic partner assignment for these ribbons was also excellent (n = 637 synapses; n = 6 midget bipolar cells; precision: 89.0% ± 5.4%; sensitivity: 93.7% ± 4.6%; Fig. 6b inset). These values demonstrate the high accuracy of convolutional nets for predicting and segmenting ribbon synapses.

By contrast with the ribbon synapses made in the IPL, ribbon synapses made by the cone pedicle in the OPL are extremely complex^52, 53^ (Fig. 1h, 1i). Post-synaptic processes can either invaginate into the face of the pedicle or occupy a basal position at the cone pedicle up to ~800 nm from the sites of transmitter release^53, 54^. We mapped all incoming cone synapses to the dendritic tips for each of six midget bipolar cells sampled. We found high precision and sensitivity for ribbon detection (n = 51 ribbons; n = 3 LM cones; precision: 92.3% ± 6.7%; sensitivity: 100% ± 0%, Extended Data Fig. 2a-2c) and for the multiple postsynaptic processes deriving from the invaginating (IMB) and flat (FMB) midget bipolar cells (n = 153 connections; precision: 98.7% ± 1.2%; sensitivity: 99.7% ± 0.6%; see Extended Data Fig. 2c right). Diffuse bipolar cells, which make more sparse connections with multiple cones, were also sampled (n = 2, DB4 and DB2; Extended Data Fig. 2d-2f), yielding similar precision and sensitivity results.

For conventional synapses, we focused on the AII amacrine cell (Figs, 4a, 5, 6c; Supplementary Data Table 1g) where an inhibitory glycinergic synapse to OFF bipolar cell axon terminals is well established^55–58^. We manually mapped all the outgoing synapses made by two neighboring AII amacrine cells and compared our ground truth data to the predicted synaptic assignments (Fig. 6c–6g). The prediction captured well the origin of synaptic output from the outer stratifying lobular appendages of the AII cell to outer-OFF bipolar cells (Fig. 6d–6g). Vesicle clouds were also detected with high sensitivity (87.5% ± 0.7%, n = 2 AII cells; false negatives were rare (Fig. 6c bottom) but relative to ribbon detection, there were more false positives, thereby reducing the accuracy of the overall synapse detection (76.0% ± 8.5%, n = 2 AII cells). With this limitation, the results show that our dataset accurately reflects synaptic connectivity.

The initial picture of the cell types and their synaptic connections in HFseg1 permitted us to ask to what degree have cells and circuits become distinctive in human vs non-human primate. Here we call attention to some examples where differences are dramatic and require further study.

### Retinal connectivity and color vision: distinctive features of the S cone circuit

Cone pedicles contact each other by short extensions (telodendria) or by distinct pedicle-to-pedicle appositions (Fig. 2b). These contacts harbor gap junctions that create electrical synapses between cones. In non-human primates, LM to LM cone contacts are abundant, but S to LM contacts are greatly reduced or absent^43, 59^: this difference may reflect a need to support color vision by isolating short from long wavelength signals^60, 61^. In human retina however S-LM cone contacts have been reported^62, 63^. We therefore mapped the full cone-cone connectome to determine whether S-LM contacts are a consistent feature of human fovea. Cone-cone contacts (n = 1141) were characterized by a thick, darkly stained region defining the contact area (see Fig. 2b insets)^64^. We found abundant S–LM cone contacts (n = 16 S cones; 5.25 ± 2.11 contacts/S-cone pedicle), indistinguishable from LM–LM cone contacts (n = 297 LM cones; 6.89 ± 3.08 contacts/pedicle). The functional significance of this species difference in cone-cone connectivity remains to be explored with biophysical modeling. Nevertheless, these data predict that in human foveal retina S-LM cone gap junctions could alter the spectral tuning of the S cone synaptic output.

A second difference in synaptic connectivity that may impact color vision occurs at the synapse between cones and horizontal cell interneurons. The horizontal cell network in the outer retina generates lateral negative feedback that underlies the antagonistic receptive field surround of cones, bipolar cells, and ganglion cells^65^. In macaque monkey retina the horizontal cell mediated surround is a key element in the circuitry that initiates red-green color vision^66–68^. In both macaque and marmoset, H1 cells make dense contacts with LM cones but largely or completely avoid contact with S cones, whereas H2 cells show some selectivity for S cones^69–71^. While the H2 cells in HFseg1 did maintain a clear preference for synapsing with S cones (Fig. 2d), we found that H1 cells also received a major input from S cones, comparable to H2 cells (H1, 43% ± 16%; H2, 57% ± 16%, n = 10 S cones). By contrast, H2 cells formed less than 2% of the horizontal cell contacts with LM cones (1.9% ± 0.1%, n = 2 LM cones; also Extended Data Fig. 4). As a consequence, human foveal H1 cells are predicted to show a trichromatic receptive field with a small S cone contribution (Fig. 2c, 2d; Extended Data Fig. 4). Taken together, the S-LM cone coupling and the combined S and LM cone connectivity of H1 horizontal cells provides new connectomic evidence for a difference between human and non-human primates in the circuits that initiate color vision^43^, and that remains to be further explored.

### Distinctive bipolar and amacrine cell circuits in HFseg1

We found differences in both bipolar and amacrine cell circuits in HFseg1 compared to expectations from macaque monkey, whereby some bipolar cell types were either greatly reduced or absent. For example, in macaque monkey non-midget or diffuse (DB) OFF bipolar cells can be divided into 4 types (DB 1, 2, 3a and 3b) but in HFseg1 we were only able to distinguish three populations that we call DB1, 2 and 3. Bipolar cell morphologies not previously described in primates were also present. One group showed amacrine-like morphology (called here outer-x and inner-x in Fig. 3a, 3b) but contained abundant ribbon synapses (20 cells, Supplementary Data Table 1f; Extended Data Fig. 6). Similar cells were discovered recently in mouse retina^11, 72^ and likely correspond to the OFF-x bipolar cells identified by transcriptomics of human fovea^13^ that show an expression profile similar to that of the midget bipolar cells. While most of these cells stratified in the outer half of the IPL (outer-x cells, n = 15, Extended Data Fig. 6a-6b), five of the cells stratified in the inner half of the IPL (inner-x cells, Extended Data Fig. 6c-6g). Another previously undescribed bipolar type showed the characteristic diffuse bipolar cell connection to multiple cones but extended axonal branches in both outer and inner IPL where it provided synaptic output to OFF-outer and ON-inner ganglion cell types (see also Extended Data Fig. 7c, 7e). We refer to this cell group as DBbroad (18 cells, Fig. 3a; Extended Data Fig. 7; Supplementary Data Table 1f). The HFseg1 volume offers a unique opportunity to further understand these novel bipolar cell populations.

For the amacrine cells the AII type (AC2) was present at the highest density of any amacrine cell population (26%, 75 cells, Figs. 4a, 4b, 5, Supplementary Data Table 1g). This cell type was recognized by its well-established morphology across species^55^ including human^40, 56, 73^. A major function of AII cells is to transfer signals from rod bipolar to cone bipolar cells, serving as conduits for rod signals to ganglion cells at scotopic light levels^74^. The presence of AII amacrine cells at such a high density in the fovea, where rods are all but absent, suggests however a function switch to a primary role in photopic spatial vision. Indeed, we observed that AII cells in HFseg1 are postsynaptic to varied cone bipolar types, including IMB cells (Fig. 5). These data support the general view that AII cells “have a day job”^56^ to provide an inhibitory pathway from ON to OFF bipolar cells under photopic conditions^74–76^ while at the same time emphasizing a specialized synaptic role for the AII cells in the human foveal midget circuit.

### Visual pathway reduction: the vertical excitatory connectome

Despite its fundamental nature the number and synaptic architecture of the visual pathways that link the human fovea to the brain is unknown. Our provisional classification of 11 pathways suggests that multiple ganglion cell types (n > 20)^32, 33, 40, 77, 78^ observed in the monkey and human retinal periphery are absent from HFseg1. This result also stands in sharp contrast to the retina of the mouse where ~40 GC types have been distinguished throughout the retina^29–31^. Our classification however had not to this point ruled out the possibility that some ganglion cell types with somas outside HFSeg1 have dendritic trees which penetrate the volume and contribute to the circuits of the inner retina. We therefore took a second approach to identifying the visual pathways in HFseg1 by mapping what we called the “vertical excitatory connectome”.

We reasoned, as others have^79^, that to generate parallel pathways to the brain each cone photoreceptor must be locally presynaptic to each cone bipolar type (for the S cone, there is uncertainty on this point, due to its more specialized role in color vision). At the next synaptic step, the output of all bipolar cell types should project to all ganglion cell types. In other words, in principle, *all visual pathways should be synaptically linked to the output of any given cone*.

To test this hypothesis, we annotated the ribbons within five cones (three LM and two S) and all postsynaptic bipolar cells associated with these cones. For each bipolar cell all synaptic ribbons and their postsynaptic GC partners were next identified (Fig. 8a–8b). Finally, we determined the relative synaptic density from a given cone via its postsynaptic bipolar cells to each identified GC type (see details in Extended Data Figs. 9 and 10; Supplementary Data Table 2).

**Fig. 7.**
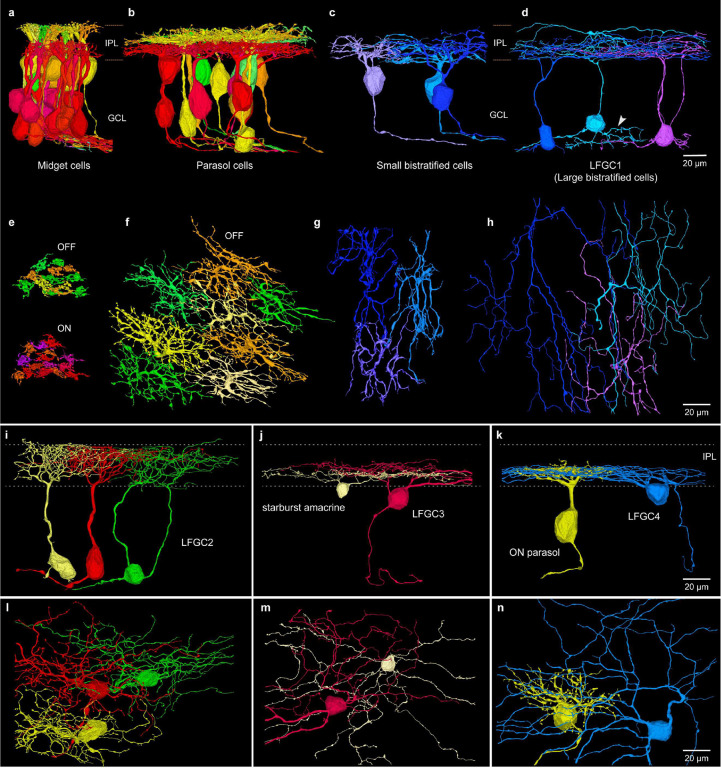
Ganglion cells. **a**. Cluster of inner-ON (red-violet) and outer-OFF (yellow-green) midget ganglion cells; dendrites stratify broadly across inner (ON) and outer (OFF) half of the IPL. **b**. Inner-ON (red-violet) and outer-OFF (yellow-green) parasol ganglion cells; dendrites stratify broadly near the center of the IPL. **c**. Small bistratified ganglion cells extend dendrites across the ON-OFF subdivision of the IPL. **d**. Large bistratified ganglion cells (LBGC1) show large dendritic field and broad stratification. White arrowhead shows branching processes arising from the cell body of unknown significance. **e-h**, Mosaic of dendritic arbors in horizontal view of the same cells shown in **a-d**. **e**. Outer-OFF (yellow-green, top) and inner-ON (red-violet) midget GC. **f**. Outer-OFF parasol cell dendrites show minimal overlap (inner-ON parasol mosaic not shown). **g**. Small bistratified cells show minimal dendritic tree overlap. **h**. LFGC1 (Large bistratified cells) show a much larger and more sparsely branching irregular tree. **i-k**. Morphology of three additional large field types. **i**. LFGC2 (Large diffuse cells) show a densely branched tree broadly stratified across the IPL. **j**. LFGC3 (Recursive bistratified cells) are narrowly bistratified near center of the IPL and tend to fasciculate with starburst amacrine cells. **k**. LFGC4 (Inner smooth monostratified cells) show large, monostratified dendritic trees costratified with parasol cells. **l-n**. Horizontal views of the cells shown in **i-k.**

**Fig. 8.**
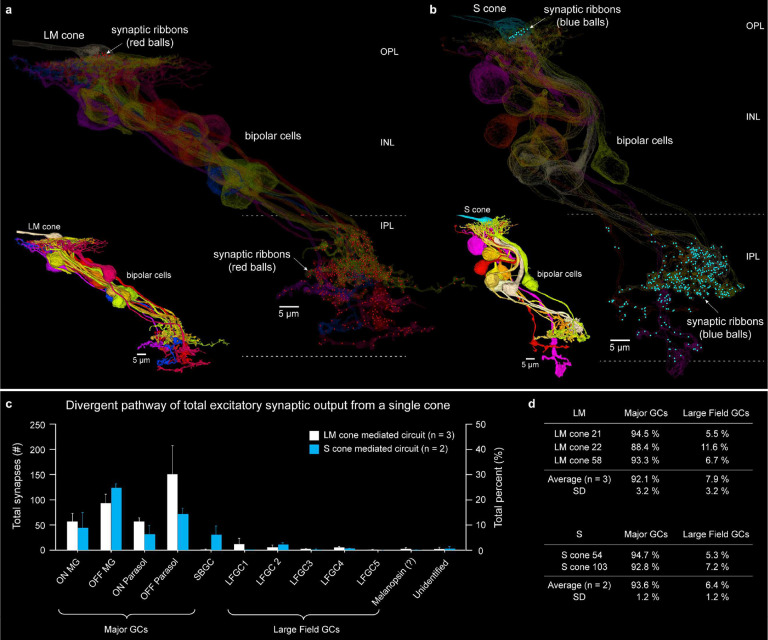
Vertical excitatory connectome. **a**. Vertical connectome for a single LM cone (cone 22, see details in Extended Data Fig. 9b). Cone pedicle and postsynaptic bipolar cells shown in silhouette. Synaptic ribbons in the cone pedicle (top, OPL) and bipolar cell axon terminals (lower, IPL) are shown as red balls. Inset shows all bipolar cells postsynaptic to this cone pedicle (outer, yellow-gold; inner, blue-violet-red). **b**. The same views for an identified S-cone (cone 103, see details in Extended Data Fig. 10b; synaptic ribbons, blue dots). **c**. Histogram shows output synapse numbers (left axis), and percent (right axis) made via bipolar cells connected to 3 LM and 2 S cones with postsynaptic GC types. Note that OFF pathway derived synapses greatly outnumber ON pathway synapses. **d**. The great majority of synapses from LM and S cones are distributed to midget, parasol and small bistratified ganglion cells (Major GCs) with all other large field types receiving 7.9% (LM) and 6.4% (S) of the synaptic output (see also Supplementary Data Table 2). Unidentified processes account for 0.5% of total synaptic output (LM) (see Supplementary Data Table 2a-2c).

We found that bipolar cells connected to the three LM cones synapsed overwhelmingly with midget and/or parasol ganglion cells (Extended Data Fig. 9), accounting for 92.1% of the total bipolar synaptic output to ganglion cells (Fig. 8c, 8d; Extended Data Fig. 9; Supplementary Data Table 2a-2c). The remaining 7.9% of bipolar synapses were directed to the large field ganglion cell types (LFGC1 - LFGC5, Fig. 7d, 7h, and 7i–7n; Extended Data Fig. 9; Supplementary Data Table 2a-2c). Thus, synaptic density corresponds well with overall cell abundance. Output to dendritic fragments of unidentified ganglion cells was rare, accounting for only ~0.5% of the total bipolar output to ganglion cells (n = 3 LM cones; 0.53 ± 0.76, n = 3 LM cones, Supplementary Data Table 2a-2c). These data align with our population analysis described above and show that, for the LM cones, diverse synaptic outputs are overwhelmingly targeted to the midget and parasol pathways.

## Discussion

### A human foveal connectome.

Our aim was to enable timely completion of a nanometer-scale connectome in the human CNS. Compared to ongoing study of a larger volume of human neocortex^19^ our foveal volume has the advantage that foveal circuitry is miniaturized and functionally complete – from receptors to ganglion cell outputs. Moreover, all cell types in the HFSeg1 volume are identified and can be related to anatomical, physiological and transcriptomic studies of human and non-human primate retina (Supplementary Data Table 1).

### Cell type profiling.

Datasets parallel to HFseg1 exist for primate central retina where cell types were clustered by gene expression profile and linked to known anatomical types by marker genes^12, 13, 15, 42, 80^. The HFseg1 volume permits a comparison of the two approaches and suggests generally good correspondence though with some unexpected differences. For example, the ratio of H1 to H2 horizontal cell types identified by transcriptomics were shown in macaque and human central retina to be ~3:1 and 7:1 respectively. By contrast the H1 to H2 ratio in HFseg1 is much higher (22:1) with H1 cells forming 96% of the horizontal cells at this foveal location. Thus, the trend towards H1 cell dominance in the transcriptomics dataset is evident but more extreme in HFseg1. A similar trend holds for other clearly identified types: for example midget ganglion cells make up ~90% of the ganglion cell population in our sample and 86% in the transcriptome; AII cells make up 26% the amacrine cells and 18% of the amacrines identified by transcriptomics. One explanation for the residual differences may be that our smaller sample represents the cone-enriched foveal center whereas the samples used for transcriptomics encompassed a much larger area (~1.5 mm, central 5 degrees). Cell type-specific density changes are extremely steep across this region so that larger spatial samples would average out such changes,

Certain cell populations previously recognized were not evident in HFseg1. For example, the recent functional, anatomical and molecular distinction made between diffuse bipolar types DB3a and DB3b in macaque monkey^81, 82^ and human retina^13, 83^ was not apparent in HFseg1. Further, we found evidence for only 19 amacrine cell types whereas molecular profiling in human retina has proposed 73 amacrine cell types^15^. These differences suggest that the relationship between cells defined by gene expression and anatomical types may not always be straightforward. A further implication is that the extreme increase in density of the midget circuit, required for high acuity and color vision, gives rise to an attendant density increase in other foveal cell types tightly linked to the midget pathway (e.g., H1 horizontal cells) and a decrease in the fovea of cell types concerned with visual processing in the retinal periphery.

### The vertical excitatory connectome.

In the retina of the mouse ~40 distinct ganglion cell populations have been recognized^29–31^. In non-human primate fewer than 20 pathways have been defined, with transcriptomic and anatomical identification in reasonable agreement^12, 32, 33^. In human fovea, only 12 pathways have been suggested by transcriptomic analysis^13, 15^ in near alignment with the current results (see also Supplementary Notes on ganglion cell types). It was nevertheless striking that in the HFseg1 dataset ~96% of the ganglion cells comprised just three anatomically distinct groups: the midget, parasol and small bistratified cells. When we manually traced the vertical connectome from a single cone to its postsynaptic bipolar cells and in turn to all postsynaptic ganglion cells we obtained a corresponding result. Thus, a fundamental, defining feature of foveal synaptic organization may be a reduction in the number of visual pathways compared to peripheral retina. A simple explanation might be the need for the midget circuit to transmit a high-fidelity signal with close to single-cone resolution, meaning that other visual pathways are not required. But there are other major changes in the connectome related to foveal function that remain to be understood. For example, rod photoreceptors are absent or greatly reduced at the foveal center, but how this might impact overall visual pathway cell type diversity and function is unknown. Similarly, the number of direction-selective visual pathways present in non-primate mammals appears greatly reduced in non-human primates^33, 84^ but the significance of this difference remains to be clarified.

### The foveal connectome and human color vision.

Most psychophysically-based models of human color vision assume the presence of two parallel color opponent channels: red vs green and blue vs yellow^109^. The red-green pathway shows opposing L vs M cone interaction and has been associated with the circuitry that feeds the midget ganglion cell^85^. However, the human psychophysically defined red-green pathway requires that a small S cone input also be incorporated^86^. The locus of this critical signal pathway remains unclear with recent data from macaque monkey implicating both retina^110^ and primary visual cortex^111^. The HFseg1 connectome shows that H1 horizontal cells, a major source of the surround in the midget circuit^51^, likely contribute antagonistic signals from all three (L, M and S) cone types, to the midget pathway, thereby offering a novel explanation for an S cone contribution to the human red-green channel. An interaction site early in S, L and M cone pathways of the human fovea is also present in unexpected S-LM cone coupling, which may work to preserve a small, high-fidelity S-cone signal in downstream pathways utilized for trichromatic color vision^87, 88^. Thus, while there is much to learn, HFseg1 already indicates that the connectome will be one route by which we come to understand the unique character of the human nervous system.

## Methods

### EXPERIMENTAL MODEL AND SUBJECT DETAILS

#### Human tissue acquisition and preparation

Human eyes (52-year-old, white male; brain dead organ donor) were acquired from the Medical University of Vienna, Vienna, Austria at the time of death by surgical enucleation. A small incision was made to give fixative access to the posterior chamber and the retina was in this way immersion fixed in 4% glutaraldehyde in 0.1 M sodium cacodylate buffer, pH7.3–7.4 at room temperature. Medical history confirmed no abnormalities of the visual system recorded and no driving limitations; the ocular status was defined medically as unremarkable at time of enucleation. Eyes were shipped in fixative to the University of Washington where the retinas were dissected and prepared for electron microscopy.

### METHOD DETAILS

#### Identification of foveal ROI, serial block-face SEM sample preparation and image acquisition

Retinal tissue was dissected under a dissecting microscope to facilitate identification of the optic disc and the center of the foveal pit as well as the major retinal meridians. We selected an ROI that extends across absolute eccentricity of ~450–650 microns (temporal retina). This location is linked to cones located ~100–300 microns from the foveal pit center due to the lateral migration of retinal circuits from the cones at the foveal center. This location encompasses much of the central 1 degree (~300 μm/deg) of visual angle (Fig. 1a–1c).

The dissected retina was then plastic embedded and prepared for electron microscopy as previously described^1^. In brief, the tissue was incubated in a 1.5% potassium ferrocyanide and 2% osmium tetroxide (OsO4) solution for 1 h. After washing, the tissue was placed in a freshly made thiocarbohydrazide solution for 20 min and then rinsed and incubated in 2% OsO4 for 30 min. Lastly, the tissue was stained en bloc in 1% uranyl acetate overnight and subsequently stained with Walton’s lead aspartate, dehydrated and plastic embedded (Durcupan, 44610, Sigma Aldrich). After evaluation of the ROI by semithin sectioning the tissue block was trimmed, gold-coated by standard methods, and mounted in an Apreo SEM Volumescope (Thermo Fisher). We cut 3028 sections at 50 nm thick for a total depth of ~150 microns, imaging an ROI (180 μm tall × 180 μm wide; 5 nm pixels) that encompassed the full vertical depth of the retina, from the Henle fiber layer (HFL) to the nerve fiber layer (Fig. 1d). The overall ROI was imaged on the blockface after each section as 25 sequential tiles (8000 × 8000 pixels or 40 × 40 μm; 75,700 total image tiles) with 10% overlap. Images were stitched within layer and aligned across layers using standard methods available in TrakEM2^2^ (NIH image plugin; Scale Invariant Feature Transform (SIFT) with affine translation) to create a good quality volume. We first evaluated this TrakEM2-aligned volume by manual reconstruction of a small number of selected cells and circuits^62^. To further develop this volume as a connectomic resource we applied deep-learning based computational approaches to realign, segment and reconstruct all cells and synaptic connections as follows^22, 38^.

#### Volume creation

##### Montaging.

Tile offsets were determined manually, then refined by template matching at 40 nm with a search window of 96 pixels. Tiles were cropped by 128 pixels to avoid edge distortions and placed to minimize the sum of least squares from the new offsets. Outliers were inspected and given manual placement. These cropped tiles were processed to create a pyramid of encodings for resolutions between 20 nm and 640 nm^89^, and then stitched to make the montaged section in two steps. In the first step, only the two neighboring tiles in Y were considered, with both neighbors being fixed. Using an online finetuner pyramid^89^ with the calculated placement as the starting point, a displacement field was generated that would warp the encoded tiles to minimize the pixel difference with the fixed tiles. The encoded tiles were then warped using this displacement field, eroded by a pixel to avoid partial pixels at the edge, blended to the fixed tiles using the mean of the pixel values in the overlap, and then passed through CLAHE enhancement to mitigate the contrast loss from the warping and blending, resulting in strips of encoded tiles where every other tile had been warped. In the second step, the strips of tiles from the first step were stitched to each other, holding every other strip fixed in a similar process. An error map was generated at both steps by computing the difference between the encodings in the overlap at 40 nm and inspected. The non-encoded tiles were cropped by 394 pixels, warped according to the composed displacement fields from the encodings, and combined to yield montaged sections.

##### Alignment.

The unaligned sections were first processed to create a pyramid of encodings^89^. A rigid transformation for the entire dataset was estimated using SIFT features^90^ extracted from the encodings at 640 nm and correspondences established between nearest and next-nearest section pairs. From the rigidly aligned encodings, displacement fields at 40 nm resolution were generated between nearest and next-nearest section pairs using an online finetuner pyramid^89^.

A convolutional net trained to detect local misalignments and/or simple mismatches caused by defects in the EM images was used to mask unreliable regions in each displacement field. In addition, 25 sections with defects that presented with non-smooth deformations were masked by hand. Pairwise displacement fields and transformation masks were then passed to a global relaxation^89, 91^. This relaxation was performed first at 2560 nm across all 3028 sections of the dataset, and subsequently in blocks of approximately 100 sections at a resolution of 640 nm, using the previously generated drift-free fields as constraints for the start and end sections of each of the 31 blocks. The resulting displacement fields were composed with the earlier rigid transformation and the final aligned image stack warped and rendered at 10 nm.

##### Segmentation.

We pretrained a convolutional net to predict affinities between neighboring voxels at 16×16×40 nm^3^ using 1,368 μm^3^ of manually annotated data derived from three mouse visual cortex datasets^92^. We used the residual symmetric U-Net architecture^93^ with modifications^94^ and trained with data augmentation previously described^95^. We trained the network for 2,000,000 iterations using four NVIDIA L4 GPUs with PyTorch data parallelism, where each GPU processed individual patches with its own model replica.

To further fine-tune this network, we manually created training data consisting of 39 μm^3^ at a resolution of 20×20×50 nm^3^. In contrast to the pretraining datasets, mitochondria were intentionally over segmented to bias boundary detection toward a more conservative approach, aiming primarily to reduce merge errors at the expense of increasing split errors. Although we initially hypothesized that this mitochondrial over segmentation would minimally impact neuron reconstruction accuracy, we observed that it occasionally resulted in split errors in scenarios where mitochondria were tightly enclosed within very thin neuronal branches. Nevertheless, due to the substantial reduction in merge errors achieved by this strategy, we considered the corresponding increase in split errors acceptable. The training setup for fine-tuning was identical to the pretraining configuration. We fine-tuned the network for 300,000 iterations.

We predicted an affinity map for the whole dataset. Dark voxels could sometimes span multiple cells and cause segmentation errors. We therefore applied a binarized mask for dark voxels with a threshold of 0.2, invalidating each dark voxel and its three incident affinities. The resulting masked affinity map was then segmented with a mean affinity agglomeration threshold of 0.25^95^. The segmentation was ingested into the Connectome Annotation Versioning Engine proofreading platform, CAVE^96^ for further analysis.

CAVE permitted proofreading to be performed by a user community accessing the web-based neuroglancer interface^95, 97^ with dynamic updating of segment ID and related synaptic connectivity. We named this segmented volume HFseg1 (Human Fovea, segmentation 1). The HFseg 1 segmentation was of high initial quality such that it was possible to identify and annotate all major cell classes within the volume and to sort these cells into populations of distinct types using the annotation tools available in neuroglancer. Current annotations have been ingested into CAVE and provide a cell type ID hypothesis that can later be tested for each proposed cell population by further analysis of synaptic connectivity. All cells are accessible at NeuroMaps (https://neuromaps.app).

Cell types were identified provisionally based on segment 3D morphology (dendritic morphology, stratification depth, and spatial regularity and interaction (mosaics) and in some cases nuclear morphology) with proofreading ongoing (> 25,000 edits) and applied thus far to correct larger obvious mergers (primarily with glial cell processes) that interfered with cell type identification and additional more detailed proofreading on circuits of interest in the current survey of cone-bipolar-ganglion cell connections. The majority of cell populations were unambiguously recognized based on previous studies of primate retina^40, 98, 99^ (e.g., the S cones, the H1 and H2 horizontal cells, the starburst and AII amacrine cell types, the midget, diffuse and blue-cone bipolar types, or the midget, parasol and small bistratified ganglion cell types); some cell types (mainly low density amacrine types) were provisionally designated subject to further proofreading and connectivity analysis. The properties and relative densities of these cell types provide the starting point from which the full synaptic connectome can be determined after proofreading by the community, and the assignment of synaptic connections to each cell in the volume. Following FlyWire^39^, we have initiated an open community of retinal neurobiologists (currently 7 labs participating) to use these tools to complete proofreading of the HFseg1 volume.

#### Synapse detection

##### Ribbon synapse detection in OPL and IPL.

A symmetric residual U-Net architecture was trained on data with a resolution of 10×10×50 nm^3^ using input patches of size 128×128×20 to generate a probability map that indicates the likelihood of each voxel belonging to a synaptic ribbon^100^. To remove less robust predictions at the boundaries, only the central 96×96×16 voxels of the input patch was used as the output. The model was trained on a total volume of 2,254 μm^3^ of manually labeled cutouts sampled from various regions across the entire dataset.

The ribbon segmentation was produced by (1) down sampling the probability map by 2×, (2) thresholding for voxels above 0.15, and (3) using 26-connectivity to extract connected components. Segments smaller than 5 voxels in a 20×20×50 nm^3^ resolution were neglected to eliminate spurious detections.

To achieve ribbon detection in the OPL segments smaller than 5 voxels in a 20×20×50 nm^3^ resolution within the bounds of the OPL were neglected to eliminate spurious detections. For each detected ribbon within a cone pedicle, postsynaptic partners were automatically identified via the following steps: (1) All neighboring segments touching the pedicle within 800 nm of the ribbon centroid were identified. (2) For each neighbor, a representative point close to the ribbon was chosen as the postsynaptic site. These constitute the set of putative synapses for each ribbon. (3) After cell identification, only putative synapses onto bipolar or horizontal cells were kept; all others were filtered out (e.g. Müller glia, neighboring cones, mitochondria). (4) Finally, for each distinct ribbon-postsynaptic cell pair, all but the shortest putative synapses were filtered out. The result was that for each ribbon in a cone, a single synaptic connection was found onto any bipolar or horizontal cell making contact with that cone pedicle within 800 nm.

For synaptic ribbons in the IPL segments smaller than 50 voxels in a 20×20×50 nm^3^ resolution within the bounds of the IPL were neglected to eliminate spurious detections. For each detected ribbon, postsynaptic partners were automatically identified via the following steps: (1) Identify the two largest profiles within a small (500 nm) window of the centroid of the ribbon which are adjacent to the presynaptic cell and are also adjacent to each other. (2) If such a pair is found, the ribbon is assumed to be presynaptic to both (i.e., a dyadic synapse). (3) If no such pair is found, then within the window, the single largest profile adjacent to the presynaptic cell is identified as the postsynaptic partner. (4) Putative synaptic connections were filtered by cell type, requiring the presynaptic cell to be previously annotated as a bipolar cell, and the postsynaptic partner to be previously annotated as amacrine, or ganglion cell. Note we did not at this time try to use a convolutional net to detect a low density of possibly ribbonless synapses.

##### Vesicle cloud detection and conventional synapse assignment in the IPL.

A symmetric residual U-Net architecture was trained on data with a resolution of 10×10×50 nm^3^ using input patches of size 128×128×20 to generate a probability map that indicates the likelihood of each voxel belonging to a vesicle cloud^100^. To remove less robust predictions at the boundaries, only the central 96×96×16 voxels of the input patch was used as the output. The model was trained on a total volume of 2,254 μm^3^ of manually labeled cutouts sampled from various regions across the entire dataset (the same cutouts as ribbon synapse detection). Manual labels were roughly annotated to mark approximate locations of vesicle clouds rather than precise boundaries.

The vesicle cloud segmentation was produced by (1) downsampling the probability map by 2×, (2) thresholding for voxels above 0.15, and (3) using 26-connectivity to extract connected components. Segments with fewer than 200 voxels were excluded, based on the range of vesicle cloud sizes encountered.

##### IPL conventional synapse assignment.

A symmetric residual U-Net architecture was trained on data with a resolution of 20×20×50 nm^3^ using input patches of size 24×24×8 to generate a probability map that indicates the likelihood of each voxel belonging to the postsynaptic cell^100^. The model was trained on 682 μm^3^ of manually annotated labeled cutouts.

The model was applied once for each vesicle cloud detected, in a window centered on the centroid of the vesicle cloud. Using the initial automatic cell segmentation, the cell with the highest mean probability within the window was selected as the putative postsynaptic partner^100^. Further filtering by cell type was applied, requiring the presynaptic cell to be previously annotated as an amacrine cell, and the postsynaptic partner to be previously annotated as amacrine, bipolar, or ganglion cell.

##### Generating the cone-cone contactome.

We identified gap junctions between cone pedicles and between cone pedicles and rods as follows. Cones and rods were manually identified and proofread to completion. Contacts were computed using abiss^101^ where a contact is considered to be a set of connected voxel faces that are shared by the same pair of segments. For each contact, we counted the number of voxel faces along each orientation (xy, xz, and yz), as well as the minimum bounding box containing all of the faces. The surface area of a contact was estimated by computing the sum of the area of all faces involved. Contacts were excluded from analysis if (1) the surface area of the contact was ≤0.04 μm^2^ (mean surface area of contact, 0.55 ± 0.3 μm^2^, n = 1141 cone-cone contacts) or (2) the center of the bounding box was more than 15 μm from the approximate center of the cone pedicle (manually identified). All remaining contacts were manually inspected with 1× coverage to determine if they corresponded to what would be manually identified as a true cone-cone contacts harboring gap junctions.

#### Proofreading, synaptic connectivity and updating with the Connectome Annotation Versioning Engine (CAVE)

As noted above we ingested synapse predictions and cell type annotations into CAVE. A critical functionality given by CAVE is that is allows collaborative users to programmatically query the up-to-date proofread connectivity graph (synaptic connectivity is updated during proofreading of each segment-cell type) as well as any changes made to the metadata (cell type IDs, descriptions, or other annotations). We used the Jupyter notebook application to execute Python code that queried the HFseg1 dataset by leveraging Python libraries that interact with the human foveal dataset, enabling access to segment IDs and synaptic assignments.

## DATA AND SOFTWARE AVAILABILITY

The HFseg1 dataset (volume, segmentation, synaptic detection and full annotation) are currently hosted by Zetta.ai (https://zetta.ai/) and all data is accessible for community proofreading by permission at NeuroMaps. The latest version of the HFseg1 dataset is also available at NeuroMaps (https://neuromaps.app).

## Supplementary Files

This is a list of supplementary files associated with this preprint. Click to download.

- SupplementaryInformation.8.6.25.pdf
- Extendeddatafigures.8.6.25.pdf
